# Is it right to estimate inter-modular connectivity from local field potentials?

**DOI:** 10.1186/1471-2202-16-S1-P93

**Published:** 2015-12-18

**Authors:** Xue-Mei Cui, Won Sup Kim, Dong-Uk Hwang, Seung Kee Han

**Affiliations:** 1 Normal College, Yanbian University, Yanji 133002, China; 2 Department of Physics, Chungbuk National University, Cheongju, Chungbuk 361-763, Republic of Korea; 3 National Institute of Mathematical Sciences, Daejon 305-811, Republic of Korea

## 

Human brains with hundreds of billions of neurons are organized in a hierarchical modular network. There have been many attempts to estimate inter-modular connectivity utilizing coherent neuronal activities of a huge number of neurons, such as the electro-encephalogram, the magneto-encephalogram, and the functional magnetic resonance imaging. Here we ask a question: Is the inter-modular connectivity estimated from the modular activities consistent with the inter-modular connectivity that could be extracted from the network connectivity of individual nodes?

**Figure 1:**
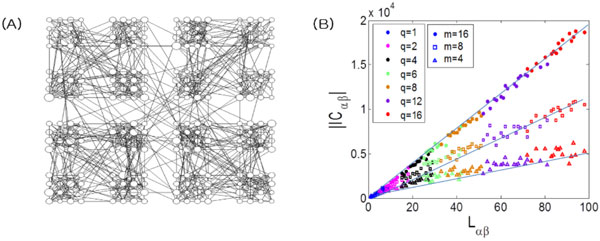
**(A) A hierarchical modular network model 256-p-q-r, where *p *denotes the number of links of one node with nodes of its lower-level module, *q *links with nodes of the rest modules in its upper-level module, and *r *links with nodes of any modules from the rest of the network**. Here we present an example of *p = 13*, *q = 4 *and *r = 1*. (B) Plot of the inverse phase synchronization index versus the number of links connecting two modules. The linear dependence between the inverse phase synchronization index and the number of links connecting two modules is shown when the local field potential is taken over *m *oscillators belonging to a subset of each module.

To answer this question, we introduce a method of estimating the inter-modular connectivity based on the analysis of inverse phase synchronization [1,2]. For coupled phase oscillators on a hierarchical modular network shown in the Figure 1(A), the local field potential corresponding to a module is defined as the mean phase of oscillators belonging to a subset of the module.

For strong coupling strength, it is shown in Figure 1(B) that the inverse phase synchronization index grows linearly with the number of links connecting two modules. This result enables us to estimate the inter-modular connectivity in various complex systems from the inverse phase synchronization index of the mesoscopic modular activities.
